# Adherence to antiretroviral therapy in young children in Cape Town, South Africa, measured by medication return and caregiver self-report: a prospective cohort study

**DOI:** 10.1186/1471-2431-8-34

**Published:** 2008-09-04

**Authors:** Mary-Ann Davies, Andrew Boulle, Tanzeem Fakir, James Nuttall, Brian Eley

**Affiliations:** 1 Red Cross War Memorial Children's Hospital and the School of Child and Adolescent Health, University of Cape Town, Cape Town, The Republic of South Africa; 2 Infectious Diseases Epidemiology Unit, School of Public Health and Family Medicine, University of Cape Town, Cape Town, The Republic of South Africa

## Abstract

**Background:**

Antiretroviral therapy (ART) dramatically improves outcomes for children in Africa; however excellent adherence is required for treatment success. This study describes the utility of different measures of adherence in detecting lapses in infants and young children in Cape Town, South Africa.

**Methods:**

In a prospective cohort of 122 HIV-infected children commenced on ART, adherence was measured monthly during the first year of treatment by medication return (MR) for both syrups and tablets/capsules. A questionnaire was administered to caregivers after 3 months of treatment to assess experience with giving medication and self-reported adherence. Viral and immune response to treatment were assessed at the end of one year and associations with measured adherence determined.

**Results:**

Medication was returned for 115/122 (94%) children with median age (IQR) of 37 (16 – 61) months. Ninety-one (79%) children achieved annual average MR adherence ≥ 90%. This was an important covariate associated with viral suppression after adjustment for disease severity (OR = 5.5 [95%CI: 0.8–35.6], p = 0.075), however was not associated with immunological response to ART. By 3 months on ART, 13 (10%) children had deceased and 11 (10%) were lost to follow-up. Questionnaires were completed by 87/98 (90%) of caregivers of those who remained in care. Sensitivity of poor reported adherence (missing ≥ 1 dose in the previous 3 days) for MR adherence <90% was only 31.8% (95% CI: 10.7% – 53.0%). Caregivers of 33/87 (38.4%) children reported difficulties with giving medication, most commonly poor palatability (21.8%). Independent socio-demographic predictors of MR adherence ≥ 90% were secondary education of caregivers (OR = 4.49; 95%CI: 1.10 – 18.24) and access to water and electricity (OR = 2.65; 95%CI: 0.93 – 7.55). Taking ritonavir was negatively associated with MR adherence ≥ 90% (OR = 0.37; 95%CI: 0.13 – 1.02).

**Conclusion:**

Excellent adherence to ART is possible in African infants and young children and the relatively simple low technology measure of adherence by MR strongly predicts viral response. Better socio-economic status and more palatable regimens are associated with better adherence.

## Background

Approximately 85% of the 2.3 million HIV-infected children under 15 years worldwide live in sub-Saharan Africa.[1] While antiretroviral therapy (ART) of children in Africa has resulted in dramatically improved survival, clinical, immunological and virological status, less than 15% of children needing ART on the continent currently receive it. [2-11] Excellent adherence is one of the most important factors in determining treatment success and preventing viral resistance, and the need for near-perfect adherence to lifelong therapy from an early age has been identified as a major challenge in the administration of ART to HIV-infected children.[10,12-15] There is concern about the extent to which this is achievable for children in resource-limited settings, particularly in the context of the rapid scale-up of pediatric treatment programs required to address the HIV burden on children in Africa.[14,16]

Research from rich countries suggests that adherence may be more complex in children compared to adults due to many factors including reliance on caregivers who may themselves be ill or may not be the child's parent, complex dosing regimens, lack of availability of pediatric fixed-dose combinations, poor drug palatability, difficulty with taking tablets/capsules and interference with daily routines. [13,17-23] Adherence estimates of 50 to 75% have been reported, well below the required 90 to 95% to achieve optimal viral suppression.[12,13,17-23]

While African adult studies show that good adherence to ART is possible despite poor social circumstances, there are limited studies in African children.[16,24-27] Health service challenges as well as individual factors such as poor socio-economic circumstances, poor literacy and the prohibitive cost of liquid drug formulations necessitating tablet/capsule administration to very young children are additional potential barriers to adherence in African children.[10,15] In Kampala, Uganda, 72% of children aged 2–18 years had adherence ≥ 95% measured with home-based unannounced pill counts, compared to 89% using 3-day self-reported adherence and 94% using clinic-based pill counts.[28] Muller et al. similarly found discrepant results using different measures of adherence in young children (median age 38 months) in South Africa.[29] Using electronic means (Medication Event Monitoring System (MEMS)) to monitor adherence, only 36% of patients achieved ≥ 95% adherence, in comparison to 91% of caregivers reporting excellent adherence on a visual analogue scale.[29] The only other published adherence studies of African children include only older children and measure self-reported adherence only, with varying results. In Côte d'Ivoire and Uganda, approximately one third of caregiver-child pairs reported missing doses and in South Africa, Reddi et al. describe 10% of children missing ≥ 3 doses during the previous month.[6,30,31] African studies thus concur with international literature that more objective measures of adherence (e.g. unannounced pill counts and MEMS) tend to be more sensitive to lapses in adherence.[21,32] However, such measures are not feasible in resource-limited settings with large-scale programs, and there is a need to determine the utility of simpler measures of adherence such as clinic-based pill-counts and self-report in predicting virological response in the African context. Furthermore, all published African studies have been conducted over short periods (≤ 3 months), mostly in older chidren, and may not reflect longer term adherence patterns in very young children.

We therefore aimed to measure the level of adherence to ART in infants and young children during the first year of treatment using both medication returned as measured at the clinic and caregiver self-report, to assess the extent to which such measured adherence predicts viral and immunological outcomes and to identify factors associated with good adherence.

## Methods

### Study design, setting and population

This was a prospective cohort study. All HIV infected children commenced on antiretroviral triple therapy between July 2002 and January 2004 (n = 122) as part of the ART program of the Red Cross Children's Hospital, a tertiary care institution in Cape Town, South Africa, were eligible and agreed to participate in the study. The ART program at the hospital began prior to the official national government ART program and was donor funded during this time. Selection criteria for commencement of ART have been described elsewhere.[2] Briefly, clinical and immunological criteria as recommended by the 2001 European treatment guidelines were followed.[33] In addition, the following limited social criteria needed to be met: having an identifiable caregiver to administer medication and attend clinic appointments; resident in Cape Town for at least 3 months; caregiver compliance with last 3 clinic appointments and caregiver willingness to comply with ongoing regular clinic attendance and monitoring. The majority of children were commenced on stavudine (d4T), lamivudine (3TC) and efavirenz (EFV – children >10 kg or >3 years) or ritonavir (RTV – children <10 kg or <3 years) as no other protease inhibitor was readily available in suitable formulation and dosage in South Africa at the time. Children were followed up with monthly clinical visits for the first year. Viral load, CD4 cell count and percentage were determined using standard laboratory methods at commencement of ART and after 1 year of treatment. The definition of undetectable viral load was <400 copies/ml. The study was approved by the University of Cape Town Research Ethics Committee [Ref: 261/2002], and all parents provided written informed consent for their and their children's participation.

### Measurements of adherence and associated factors

Clinical and socio-demographic characteristics at commencement of ART were determined by the clinician treating each child and recorded on standardized data collection forms. Children were retrospectively re-staged according to medical record information using the WHO 4-stage clinical classification for the purpose of this analysis.[15] Weight-for-age, height-for-age and weight-for-height z-scores were calculated with EpiInfo 2000, version 1.0 (Division of Surveillance and Epidemiology, CDC, Atlanta, Georgia).

### Measurement of adherence by medication return (MR)

At every monthly visit for one year, caregivers were requested to return all empty medicine containers and unused medication. A dedicated program pharmacist measured the amount of unused medication volumetrically for syrups/solutions and by pill count for tablets/capsules. The percentage adherence for each antiretroviral medication was calculated by dividing actual use (determined from returned containers and unused medication) by expected use (determined from the previous month's script).

A composite measure of annual average percentage adherence by MR was calculated by determining the arithmetic mean of the percentage adherence for each drug at each monthly visit. For caregiver-child pairs who did not return medication at every visit as requested, the annual average percentage adherence was calculated using the number of months for which medication was actually returned as the denominator when determining the arithmetic mean (per protocol analysis). Sensitivity analysis was additionally performed assigning adherence <90% for months in which medication was not returned and recalculating annual average percentage adherence for children with missing medication returns. A small amount of extra medication (in excess of what was prescribed) was issued at each visit so that patients would not be without medication if drugs were spilled or additional doses required due to vomiting or spitting out. For a number of medication returns, therefore, more drug was used than prescribed (i.e. adherence >100%) so adherence was capped at 100% per return when calculating annual average adherence. An uncapped annual average MR adherence was also calculated for some of the analyses.

### Measurement of adherence by questionnaire

A standardized interview was administered by the treating clinician to each caregiver after the child had completed 3 months of ART. The interview script was based on Pediatric Aids Clinical Trials Group (PACTG) adherence questionnaires modules 1 and 2 and assessed caregiver's ability to accurately describe the ART regimen, recall of missed doses in the past 3 days, difficulties experienced with giving medication and beliefs about ART.[34,35] Based on reported missed doses, children were classified as not fully adherent (NFA) if ≥ 1 dose was missed in the previous 3 days. Interpreters were used so that interviews were conducted in the language of the caregiver's choice.

### Analysis

All statistical analysis was done using Stata (version 10) (Stata Corporation, College Station, Texas, USA). The effects of low annual average (capped) MR adherence, average (capped) MR adherence in the month immediately preceding viral load measurement and reported NFA on viral load suppression and immunological response were determined using logistic regression models adjusted for other determinants of outcome. MR adherence was dichotomized as ≥ 90% or <90% as this threshold explained the largest amount of variability in the outcome. Univariate and multivariate analysis of the association between demographic, social and clinical factors as well as experiencing problems with giving medication and annual average MR adherence ≥ 90% were examined using Wilcoxon rank sum (Mann-Whitney), Student's t-test, chi^2 ^or Fisher's exact tests and logistic regression models as indicated. Agreement between MR adherence <90% and caregiver reported NFA was calculated using the kappa statistic.

All multivariate models were built by sequentially adding the next most significant predictor variable from the univariate analysis, and variables with a p-value <0.1 after adjustment for those already included in the model, or that changed the OR for variables included in the model by more than 10%, were retained. Since variables reflecting severity of illness (WHO stage, weight-for-height z-score, CD4 percent and absolute count and log viral load) were highly correlated with one another, only the single most predictive variable, i.e. weight-for-height z-score, was included in the multivariate models for virological and immunological outcome. Similarly, only one measure of socio-economic status (formal housing, access to water and electricity, access to a refrigerator and employment) was used at a time in each model, i.e. access to water and electricity. Only socio-demographic factors and regimen were included in building the multivariate model of MR adherence ≥ 90% as the sample size did not allow for inclusion of clinical factors as well. P-values for all statistical analyses are reported exactly with no particular cut-off used to define significance.[36]

At the time that the study was designed, few data on paediatric adherence from resource-constrained countries were available on which to base sample size calculations. The change from donor-funded ART to the government ART program necessitated ending of the study as the government program did not fund adherence monitoring. By this stage the accumulated sample size was sufficient to detect a 50% reduction in proportion with viral suppression in those with MR adherence <90% with 90% power, assuming that 75% of children had MR adherence ≥ 90%.

## Results

**Figure 1:**
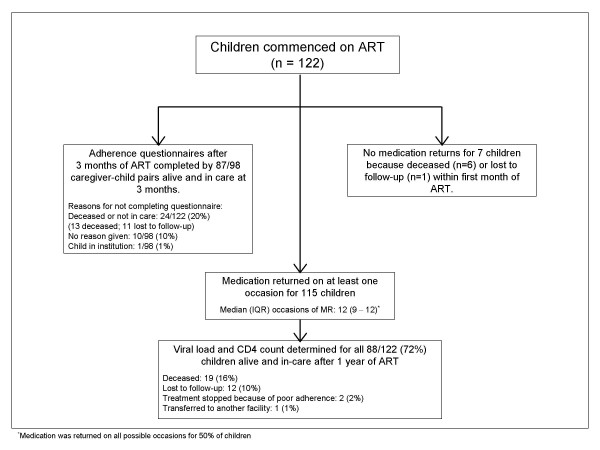
Profile of study.

Medication was returned on at least one occasion for 115/122 (94%) children who commenced ART with the remaining children deceased (n = 6) or lost to follow-up (n = 1) before their first follow-up visit (figure 1). After 1 year, 88 children were alive and remained in care on ART. Table 1 shows the demographic and clinical characteristics and ART drugs prescribed at baseline. Children were young with a median age (IQR) of 37 (16 – 61) months. Although overall socio-economic status was poor with high levels of caregiver unemployment (73%) and informal housing (49%), most caregivers (88%) had at least secondary education and the majority of children (80%) were from households with access to water and electricity.

**Table 1:** Social, demographic and clinical characteristics at start of ART according to annual average MR adherence.

**Variable**	**All children (n = 122)**	**Adherence ≥ 90% (n = 91) (79%)**	**Adherence<90% (n = 24) (21%)**	**p-value**
Median age (months) (IQR)	37 (16 – 61)	37 (16 – 57)	49 (20 – 72)	0.49*
Gender				
Female (%)	52 (43)	39 (43)	12 (50)	0.53^†^
WHO stage				
4 (%)	54 (44)	34 (37)	14 (58%)	0.06^†^
Mean weight-for-age z-score (sd)	-2.02 (1.54)	-1.82 (1.55)	-2.41 (1.37)	0.09‡
Mean height-for-age z-score (sd)	-2.71 (1.36)	-2.66 (1.36)	-2.74 (1.33)	0.78‡
Mean weight-for-height z-score (sd)	-0.56 (1.44)	-0.31 (1.34)	-1.08 (1.42)	0.02‡
Median CD4 percent (IQR)	11.1 (6.9 – 15.0)	11.4 (8.0 – 15.1)	10.1 (4.2 – 14.3)	0.18*
Median CD4 count (IQR)	556 (242 – 908)	592 (273 – 938)	518 (105 – 758)	0.23*
Median Log Viral load (IQR)	5.57 (5.15 – 6.08)	5.44 (5.11 – 6.08)	5.77 (5.30 – 6.06)	0.55*
Primary caregiver				
Mother (%)	107 (88)	79 (87)	21 (88)	0.72^§^
Not mother (%)	12 (10)	9 (10)	3 (13)	
Unknown (%)	3 (2)	3 (3)	0 (0)	
Median age of caregiver (IQR)	29 (26 – 32)	30 (26 – 32)	28 (25 – 35)	0.58*
Father provides financial support				
Yes (%)	48 (39)	37 (41)	9 (38)	0.8^†^
No (%)	70 (57)	51 (56)	14 (58)	
Unknown (%)	4 (3)	3 (3)	1 (4)	
Caregiver has secondary education				
Yes (%)	107 (88)	83 (91)	18 (75)	0.03^†^
No (%)	11 (9)	6 (7)	5 (21)	
Unknown (%)	4 (3)	2 (2)	1 (4)	
Caregiver employed				
Yes (%)	27 (22)	23 (25)	4 (17)	0.37^†^
No (%)	89 (73)	64 (70)	19 (79)	
Unknown (%)	6 (5)	4 (4)	1 (4)	
Caregiver/child receives social grant				
Yes (%)	69 (57)	55 (60)	11 (46)	0.16^†^
No (%)	51 (42)	34 (37)	13 (54)	
Unknown (%)	2 (2)	2 (2)	0 (0)	
Formal housing				
Yes (%)	62 (51)	50 (55)	8 (33)	0.06^†^
No (%)	60 (49)	41 (45)	16 (67)	
Access to water and electricity				
Yes (%)	97 (80)	76 (84)	15 (63)	0.024^†^
No (%)	25 (20)	15 (16)	9 (38)	
Access to working refrigerator				
Yes (%)	85 (70)	66 (73)	12 (50)	0.036^†^
No (%)	37 (30)	15 (16)	12 (50)	
Medication				
d4T (%)	110 (90)	81 (89)	22 (92)	1.00*
AZT (%)	11 (9)	8 (9)	3 (13)	0.70*
3TC (%)	117 (96)	88 (97)	22 (92)	0.28*
ddI (%)	4 (3)	3 (3)	1 (4)	1.00*
Efavirenz (%)	67(55)	55 (60)	10 (42)	0.08^†^
Ritonavir (%)	55 (45)	36 (40)	14 (58)	0.10^†^

*Wilcoxon rank sum test

‡Student's t-test

^†^Chi^2 ^test

^§^Fisher's exact test

### Annual average MR adherence

**Figure 2:**
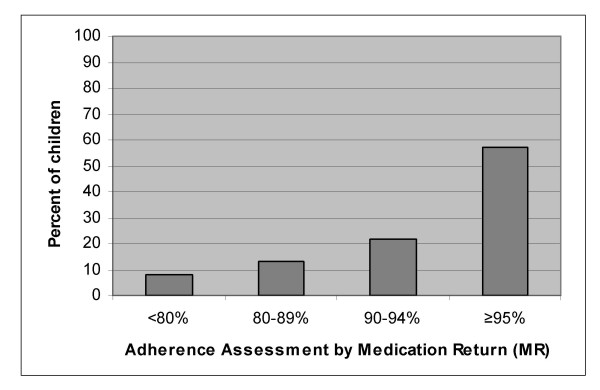
Annual average MR adherence (n = 115).

**Figure 3:**
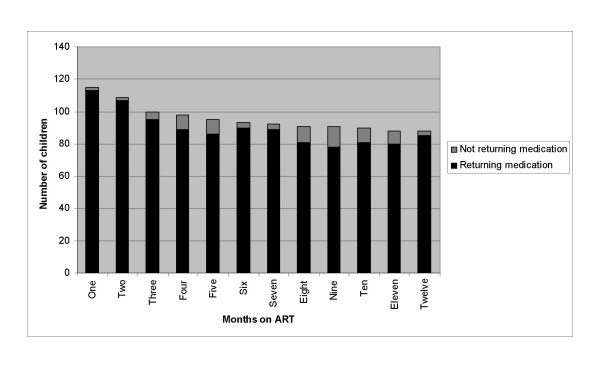
**Number of children in care at the end of each month on ART**. Total number of children in care at the end of each month divided into those returning and not returning medication

**Figure 4:**
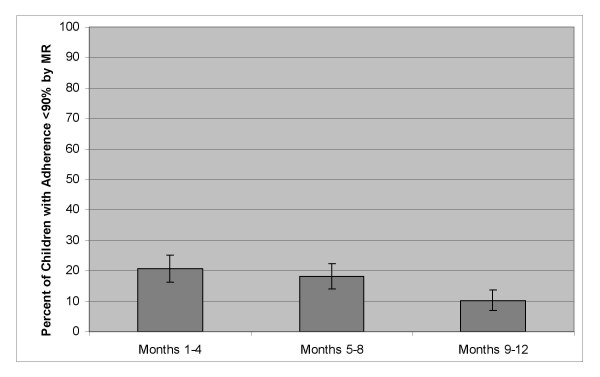
**Changes in proportion with MR adherence <90% during the first year on ART**. Only children who remained alive and in care for entire first year of treatment (excludes children deceased or LFU; n = 88).

A total of 91/115 (79%) children achieved annual average MR adherence ≥ 90% with 73% of these having adherence ≥ 95% (figure 2). Only 9 (8%) children had average adherence for the full year below 80%. These percentages did not change substantially if adherence <90% was assigned for missing returns in children not returning medication on all possible occasions. The number of children remaining in care at each month and the proportion returning medication as requested is shown in figure 3. There was no change in the proportion of children returning medication over time (p = 0.17). Among only those children who remained alive and in care for the entire first year of treatment (excluding those deceased and LFU; n = 88), the proportion with adherence <90% decreased over time with an OR for having low adherence of 0.91 (95% CI: 0.87 – 0.96; p = 0.000) for each additional month (figure 4). Annual average MR adherence <90% was more likely among the 34/115 (30%) child-caregiver pairs who failed to return empty medication containers/unused medication at more than one follow-up visit (OR = 4.97; 95% CI:1.92 – 12.87; p = 0.001).

### Relationship between MR adherence and viral/immunological outcomes

Undetectable viral load was achieved in 62/80 (78%) children with annual average MR adherence ≥ 90% compared to 2/8 (25%) of children with lower adherence (OR = 10.3; 95% CI: 1.92 – 55.7; p = 0.005). In univariate analysis, other factors significantly associated with viral suppression were less wasting as reflected in higher weight-for-height z-score, less severe disease (WHO stage 2 or 3 vs WHO stage 4) and being on a non-ritonavir containing regimen (Table 2). Since nutritional status is an important determinant of WHO stage, only weight-for-height z-score and regimen were included in building an adjusted logistic regression model for the relationship between adherence and viral suppression. After adjustment for wasting, regimen was no longer associated with viral load outcome, however adherence ≥ 90% remained an important covariate associated with viral suppression (Adjusted OR = 5.5 [95%CI: 0.8–35.6], p = 0.075). Sensitivity analysis was performed by recalculating average annual adherence for caregiver-child pairs who did not return medication for one or more months by assigning adherence <90% for months in which medication was not returned, and there remained a significant univariate association between MR adherence ≥ 90% and undetectable viral load, and a strong trend towards an association after adjustment for baseline weight-for-height z-score. There was no association between MR adherence in the month or 2 months preceding viral load measurement and viral suppression in either univariate analysis or after adjustment for baseline weight-for-height z-score. Similarly no association was found between the proportion of visits in which caregivers failed to return medication and viral suppression in either univariate or adjusted analyses.

**Table 2:** Univariate and multivariate factors associated with undetectable viral load (n = 88)

**Variable**	**Unadjusted OR**	**95%CI**	**p-value**	**Adjusted OR**	**95%CI**	**p-value**
Female gender	0.91	0.36 – 2.35	0.86	^†^		
Age at treatment start (months)	1.00	0.99 – 1.01	0.81	^†^		
WHO stage 2&3 (vs WHO stage 4)	2.80	1.07 – 7.35	0.04	*		
Weight-for-age z-score	1.29	0.94 – 1.77	0.12	*		
Height-for-age z-score	1.07	0.74 – 1.54	0.71	*		
Weight-for-height z-score	1.94	1.27 – 2.96	0.002	1.8	1.15 – 2.80	0.01
CD4 percent	1.04	0.97 – 1.12	0.27	*		
CD4 absolute (cells/l)	1.44	0.54 – 3.84	0.47	*		
Log viral load	0.87	0.48 – 1.60	0.66	*		
Ritonavir-containing regimen	0.33	0.13 – 0.87	0.03	^†^		
Annual average MR adherence ≥ 90%	10.30	1.92 – 55.67	0.005	5.48	0.84 – 35.58	0.075

*Not included in building multivariate model

^†^Not significantly associated with viral suppression after adjustment for weight-for-height z-score

The median (IQR) changes in CD4 percent and absolute count were 10.1% (5.7% – 15.2%) and 393 cells/μl (113 – 654) respectively. There was no association between annual average MR adherence ≥ 90% and either CD4 percent at 1 year, or change from baseline CD4 percent or absolute count over 1 year in either univariate analysis or models adjusted for other determinants of immunological response.

### Excess MR Adherence

Using the uncapped annual average MR adherence, 47/115 (40.9%) of children had adherence >100%. This was more common among children taking ritonavir (30/50 [60%]) compared to those on efavirenz (17/65 [26%]; p = 0.000). Adherence >100% was also more common in those 2 years of age or less (28/37 [75.7%]) compared to older children (19/78 [24.4%]; p = 0.000). This age-related difference in excess adherence was maintained after stratifying for ritonavir-based regimen (p = 0.02 and 0.05 for those with ritonavir-based and efavirenz-based regimens respectively).

### Questionnaire responses

Of 98 children alive and in care after 3 months of ART, 87 (90%) completed adherence questionnaires (figure 1). Annual average MR adherence ≥ 90% was more common in those who completed questionnaires (59/87 [68%] vs 4/11 [36%]; p = 0.051).

### Reported missed doses

NFA (missing ≥ 1 dose in the previous 3 days) was present in 12/87 (13.8%) children, and was more common in those whose MR adherence for that month was <90% (7/22 [31.8%]) vs 5/62 [8.1%]; p = 0.006). Nevertheless, the sensitivity of NFA for poor MR adherence in the preceding month was only 31.8% (95% CI: 10.7% – 53.0%) and agreement between the two measures was only slightly better than that expected by chance (κ = 0.278; p = 0.003). There was no association between NFA and viral response at 1 year (p = 0.965). Although 38/87 (43.7%) of caregivers were unable to describe how to give their child's ART regimen exactly, this was not associated with either NFA or MR adherence <90% (p = 0.88 and p = 0.68 respectively).

### Experience with giving medication

A notable number of caregivers (33/87 [38.4%]) experienced problems with giving ART medication. Poor palatability of medication was the most common problem (21.8% of caregivers), with 68% of these being attributed to ritonavir. Change in daily routine was a problem for 12.6% of caregivers. Experiencing problems with giving medication did not affect reported (OR = 2.13; 95%CI: 0.59 – 7.65; p = 0.32) or MR adherence (OR = 0.61; 95%CI: 0.22 – 1.65; p = 0.32) in the month in which problems were reported, but was associated with annual average MR adherence <90% (OR = 3.07; 95% CI: 0.91 – 10.38; p = 0.06).

Most (65/87 [74.7%]) caregivers used at least one method to assist with remembering and giving medication. The most commonly used aids were activities of daily living reminders (35 [40%]) and treatment partners (23 [26%]). While the vast majority of caregivers (81 [93%]) believed that ART medication was improving their child's health, a significant number (24 [28%]) were unsure or believed that their children would not deteriorate if ART was stopped. Caregiver beliefs did not influence adherence by any measure.

### Determinants of annual average MR adherence ≥ 90%

Socio-demographic variables most strongly associated with annual average MR adherence ≥ 90% on univariate analysis were caregivers having secondary education and household access to water and electricity as well as a refrigerator (tables 1 and 3). Having secondary education was not significantly associated with any of the indicators of socio-economic status. Being on an efavirenz-based regimen was less strongly associated with good adherence. Secondary education of the caregiver and household access to water and electricity were both independent socio-demographic predictors of annual MR adherence ≥ 90%, while taking ritonavir was negatively associated with MR adherence ≥ 90%.

**Table 3:** Factors associated with annual average MR adherence ≥ 90%. (n = 115)

**Variable**	**Unadjusted OR**	**95% CI**	**p-value**	**Adjusted OR**	**95% CI**	**p-value**
Access to water and electricity	3.04	1.12 – 8.22	0.028	2.65	0.93 – 7.55	0.069
Formal housing	2.44	0.95 – 6.27	0.064	*		
Access to working refrigerator	2.64	1.04 – 6.65	0.039	*		
Secondary education (>Std 5/Grade 7)	3.84	1.06 – 13.98	0.041	4.49	1.10 – 18.24	0.035
Taking ritonavir	0.44	0.18 – 1.11	0.084	0.37	0.13 – 1.02	0.054

*Not included in multivariate model as only one measure of socio-economic status (access to water and electricity) used.

## Discussion

This study extends to infants and young children the finding that good adherence to ART in Africa is achievable with nearly 80% of children obtaining average MR adherence ≥ 90% over the first year of ART. [16,25,26,28] This is at least as good as pediatric adherence in rich countries.[13,17-22] This excellent adherence occurred despite nearly 40% of caregivers experiencing subjective difficulty with administering medication. Secondary education, access to water and electricity and a non-ritonavir based regimen were all independently associated with better adherence. This study demonstrates that although clinic-based medication measures may not be as sensitive as unannounced home-based measures or MEMS monitoring to detect poor adherence, they are still strongly predictive of virologic response. Annual average MR adherence ≥ 90% was associated with a greater than 5 fold increased likelihood of suppressing viral load at the end of the year.

The excellent adherence seen in our study may, however, not be representative of current pediatric adherence in Africa as ART is scaled up. These were the first children to receive ART at our tertiary care institution and many were well-known to the HIV service as adherent with other medications and clinic visits. In addition, the social criteria used to determine ART eligibility may have further selected those patients more likely to be adherent. Furthermore, treatment was donor-funded in the context of no access to ART through government health services so caregivers may have felt that ART was a privilege. Moreover, regular adherence monitoring may have enhanced adherence. While the halving of the proportion of children with MR adherence <90% during the year supports this, it is a problem inherent to all adherence studies. In addition, the actual estimate of adherence may be inflated by use of clinic-based medication measures. The finding by Muller et al.[29] of much lower adherence using MEMS caps in a recent post-national ART roll-out study in a similar setting to ours supports the likelihood of both particularly good adherence in our cohort, and of adherence being over-estimated by our method of measurement. Nevertheless, the strong association with viral suppression in our study indicates at least reasonable accuracy of adherence measurement.

In contrast, reported NFA showed no relationship to viral outcome and was only 32% sensitive for MR adherence <90%. While the failure to find an association with viral suppression may well be due to the fact that reported adherence was only measured once long before viral load measurement, the tendency for caregivers and patients to over-estimate adherence is well established.[15,23,28,29,32] In our study, this may have been exacerbated by interviews being conducted by clinicians to whom caregivers might be reluctant to admit to missing doses, particularly in the context of limited access to ART. This, however, reflects clinical practice.[15], and any attempts to develop a screening tool for poor adherence in busy roll-out clinics must take into account under-reporting of non-adherence to clinicians. In this study, the additional measuring of adherence by medication return should have, at least in part, mitigated against under-reporting.

Interestingly we found no association between MR adherence in the month or 2 months immediately prior to viral load measurement, in contrast to the strong association with average MR adherence over the whole year. Other studies have found strong associations between short-term adherence and viral suppression immediately thereafter. [12,29] Having a single viral load measurement only after a full year on treatment made it impossible to examine the effect of short-term fluctuations in adherence on viral outcome. The small sample in this study may also have reduced its power to show such an association particularly as a far smaller number of children than expected had poor adherence. In addition, the sample size limited the number of predictor variables that could be used in analytic models. We were only able to include a single indicator of disease severity in the model of viral suppression and only able to include socio-demographic and regimen factors as predictors of MR adherence ≥ 90%.

It is notable that nearly 40% of caregivers experienced problems with giving ART, and that this adversely affected adherence over the whole year. Poor palatability of medication was the most common problem reported, especially in those taking ritonavir. This problem is unique to infants and very young children taking liquid formulations of drugs, and concurs with international studies.[18,22] A limitation of our study is that drug formulation was not recorded so its effect on adherence could not be examined. However, the finding that a greater proportion of children under 2 years of age (who would all be taking liquid formulations) had adherence >100% suggests that repeat dosing of syrups/solutions is frequently necessary, placing an additional burden on caregivers.

It is further noteworthy that MR adherence results in the ritonavir group appear paradoxical: taking ritonavir was both negatively associated with (capped) MR adherence ≥ 90%, and positively associated with (uncapped) MR adherence >100%. The excess adherence is explained by frequent need for repeat dosing. However poor adherence in the ritonavir group when measures are capped indicates that there is not always compliance with frequent dosing with an unpalatable drug ultimately impacting negatively on adherence. Indeed the poor adherence in the ritonavir group was attributable to poor adherence to ritonavir alone, with adherence to the other two drugs in the regimen being acceptable (data not shown). Although ritonavir is no longer used as a single third agent in ART regimens, Kaletra^® ^(lopinavir/ritonavir) is also unpalatable and is recommended in the South African national guidelines as part of the first-line regimen in children under 3 years of age.[37] Moreover, drugs other than ritonavir accounted for nearly a third of palatability problems. The need for pediatric-friendly formulations of ART cannot be over-emphasized.

While some studies have suggested that older children are more likely to be non-adherent, we found no relationship between age and adherence.[20,30] However, in our study the median age was young (36 months) with few children approaching adolescence, so its potential negative effect could not be determined. Nevertheless, it should be noted that the age of children commencing ART at our institution has decreased since this initial program, with the majority of children now being less than 2 year old.[8] Our findings may therefore not be applicable to our current patient cohort, and there is a need for further research into adherence in very young infants in Africa.

While previous research in Africa has found little impact of socio-economic status on adherence, our study suggests that better caregiver education and socio-economic status are both strongly independently associated with better adherence. [16,25,27,29] Caregivers with secondary education and those with access to water and electricity are 4.5 times and 2.7 times more likely to have adherence ≥ 90% respectively. The lack of association between caregiver education and any of the indicators of socio-economic status emphasizes that these both impact on adherence and one is not simply a surrogate marker of the other. Compared to adult ART, administering medication to children, particularly infants, is more complex and requires exact measurement of dosages, often to a fraction of a milliliter, and compliance with stringent storage requirements. In addition, unlike adults where dosage remains constant over a long period, dosages for children change frequently due to their rapid growth. Although we did not examine the effect of complex dosages and dosage changes on MR adherence, the positive impact on adherence in children of better education and socio-economic status of their caregivers is not surprising. However, the failure to find an association with caregiver's ability to describe a regimen and adherence suggests that it is the caregiver's overall education that impacts more on treatment adherence than treatment literacy per se.

## Conclusion

This study demonstrates the potential for caregivers of African young children to achieve adherence comparable with that of wealthier countries. The association between MR adherence and viral response attests to the value of a relatively simple low technology tool for measuring adherence i.e. clinic-based medication return for detecting lapses in adherence. Nevertheless, repeated measurements of medication returned, particularly of syrups/solutions, are not feasible in most large program scale-up settings and the poor sensitivity of low reported adherence for low MR adherence highlights the need to develop practical easy-to-use reliable screening tools to detect children in whom more intensive adherence monitoring is indicated. [30] The negative impact of problems experienced with giving ART, unpalatable drugs, poor caregiver education and socio-economic status on adherence in this study underscores the need for more pediatric-friendly drug formulations as well as the importance of supporting caregivers in providing ART to children.

## Competing interests

The authors declare that they have no competing interests.

## Authors' contributions

MD conceived of the study, participated in its design and co-ordination, performed statistical analysis and drafted the manuscript. BE participated in the design and co-ordination of the study and advised on analysis. AB provided advice on statistical analysis. JN participated in the design and co-ordination of the study. TF carried out all medication measurements. All authors read and approved the final manuscript.

## Pre-publication history

The pre-publication history for this paper can be accessed here:


